# Normal development of context processing using the AXCPT paradigm

**DOI:** 10.1371/journal.pone.0197812

**Published:** 2018-05-31

**Authors:** Nicola Riccardo Polizzotto, Tanisha Hill-Jarrett, Christopher Walker, Raymond Y. Cho

**Affiliations:** 1 Department of Psychiatry and Behavioral Science, University of Texas Health Science Center at Houston, Houston, Texas, United States of America; 2 Department of Psychiatry, University of Michigan, Ann Arbor, Michigan, United States of America; 3 Department of Psychiatry and Behavioral Science, Baylor College of Medicine, Houston, Texas, United States of America; Waseda University, JAPAN

## Abstract

Context processing involves a flexible and continually updated representation of task relevant information and is a core aspect of cognitive control. The expectancy AX Continuous Performance Test (AXCPT) was designed to specifically measure context processing and has been widely applied to elucidate mechanisms of cognitive control and their impairments in conditions such as aging and schizophrenia. Here we present a large-sample, cross-sectional study of context processing aimed at characterizing its normal development from childhood to early adulthood (8 to 22 years old). We track the age-related changes in the standard AXCPT performance measures and also investigate their validity using detailed data-driven method. We show how critical maturational changes in context processing can be validly tracked from mid-adolescence onward with increasing reliance on preparatory, proactive strategies well into early adulthood. However, the early maturation from childhood into adolescence showed a sharp, two-fold discontinuity: while standard measures provide partially conflicting results suggesting an early worsening of proactive strategies, further analyses do not support their validity during this period. Our findings advocate the existence of multiple preparatory strategies that cannot be captured by indices that assume a simple dichotomy of proactive vs. reactive strategies. When evaluating context processing differences over development or in clinical populations, we advocate the explicit testing of the assumptions underlying standard AXCPT indices through complementary data-driven methods.

## Introduction

In navigating complex, dynamically changing environments, actions are not inherently right or wrong, but rather viewed by their context appropriateness. Mounting appropriate actions can be challenging when habitual, prepotent responses are inadequate in meeting current goals. In such situations, cognitive control is engaged for goal-appropriate behavior, relying on *context processing* (CP), the active maintenance of updated information regarding the relations between stimuli and responses (more recently, referred to as the goal maintenance component of working memory [1]).

While CP can be assumed to play a role in any task, the expectancy AX continuous performance test (AXCPT) was designed to specifically index such a function [2]. Theoretically motivated summary behavioral measures have been largely used to index CP estimates, while controlling for confounds [3].

As in other continuous performance tasks, subjects are instructed to detect targets and nontargets within a stream of presented letters. Targets are defined as the letter X, but only when it follows the letter A; conversely, X following non-A cues are nontargets. In other words, after any non-A letter (referred to as B cues), only nontarget responses are appropriate. Since AX pairs are presented far more frequently than any other combination of letters, for infrequent trial types it is critical to update the information about the cue—i.e. CP. During such trials, habitual target responses need to be overcome, increasing demands for cognitive control. Trials that share similarities with AX can be particularly misleading and provide a contrast to interpret the overall ongoing strategy. AX performance can be contrasted with BX trials in order to index context-specific recognition of X-targets [2]. In BX trials responses are facilitated if one readily prepares a nontarget response after the B cue onset, but responses can be incorrect and slow if cue information is retrieved only when the X probe is presented. However, in trials where A is not followed by X (AY) the cue-driven expectancy for the more frequent target response makes such proactive strategy less advantageous than a reactive, probe-driven strategy. Accordingly, it is common to predict that individual variabilities along the proactive vs. reactive continuum would have divergent effects on AY and BX trials, and to use the difference in performance in such trials (AY-BX) as a sensitive index of the control proactivity [4].

The task has shown discriminative power in differentiating populations with putative differences in CP. Schizophrenia research has employed the AXCPT as one of the tools of choice for evaluating cognition [5]. The use of standard AXCPT indices to capture CP impairments, together with formal modeling [6], have served as basis for providing a parsimonious account of cognitive disturbances in this disorder as well as in other populations [4]. Such a theory-driven account indicates that the AXCPT could provide critical insights regarding the nature and time course of CP maturation over childhood and adolescence. This would be of particular interest given the importance of this age range in neurodevelopmental disorders such as schizophrenia and the appeal of a developmental dissection to the end of unveiling the architecture underlying adult performance. Furthermore, prefrontal cortex—which both theoretical and empirical studies suggest to play a key role in CP [2,6,7]–has a complex developmental trajectory characterized by critical changes through adolescence and a prolonged maturation well within early adulthood [8].

Prior developmental AXCPT studies have provided a collection of pairwise comparisons between age groups. Collectively, they appear to trace a protracted trajectory of age-related CP changes with increase in the reliance on proactive strategies from childhood into early adulthood. When compared with 3.5 year-old children in a simplified version of the AXCPT, 8 year-olds showed an adult-like proactive reliance on context information [9]. Other studies [10–12] employed a more standard AXCPT in older cohorts and sparsely sampled from late childhood to young adulthood. For instance, 12 year-olds were shown to have worse CP compared to 22 year-olds [11], but better than 9 year-olds, as shown in a separate study from the same authors [12]. However, the two studies reported very different performance for 12 year-olds—possibly due to the use of diverse task parameters—which questions the comparability and interpretation of such observations. Further, none of these studies controlled for demographics or intellectual capabilities, which are likely to bias and confound comparisons of subjects sampled from schools (6^th^ grade vs college, [11]) or special populations (incarcerated vs community, [10]).

In the current large, cross-sectional study, we have addressed many of the major limitations in prior studies and derived the trajectory of CP maturation from childhood to young adulthood. First, we have employed a large sample (n = 186), allowing a dense sampling over a broad age range (8–22 years old). We have also employed a uniform task paradigm and controlled for demographic and cognitive profiles to ensure valid comparison across age-groups. Furthermore, in order to critically evaluate standard interpretations and the discriminative power of the AXCPT, we conducted a developmental dissection of AXCPT performance with a two-pronged approach, complementing theory-driven indices with a more data-driven approach including correlation and chronometric analyses.

We show that standard analysis using model-based indices provide evidence for a protracted, but non-monotonic maturation of performance, suggesting unexpectedly that children and adults equally rely on proactive strategies. However, our data-driven analyses distinguish such age ranges as their responses show critical differences in the pattern of relations across trial types and chronometric profiles. The findings challenge accounts that explain variability solely along a unique proactive vs. reactive dimension by advocating the existence of multiple preparatory strategies. Such analyses also suggest that AXCPT performance patterns are in agreement with standard CP assumptions only from mid-adolescence onwards. We discuss how the whole performance trajectory is better understood by addressing critical points about CP and its relation with the development of other cognitive control functions.

## Methods

### Participants

We assessed 186 healthy individuals aged 8–22 years old, binned in five age groups: 8–10 years (n = 45), 11–13 years (n = 34), 14–16 years (n = 38), 17–19 years (n = 35), and 20–22 years (n = 34). Age bins were matched by gender (50% females), handedness (7% left handed), parental socioeconomic status, ethnicity (68% Caucasian). Subjects were recruited through advertisement in community and hospital settings. Potential participants were excluded using the MINI for having a history of DSM IV Axis I or developmental disorders diagnosis, or a first-degree relative with a history of psychosis. All participants completed IQ assessment (Wechsler Abbreviated Scale of Intelligence, [13]). All IQ scores were in the normal range and balanced across groups. Fifteen subjects were excluded as outliers from analysis (over ± 4 standard deviations from the age bin mean values in any trial wise measure). Age bins did not differ in the frequency of outliers (χ^2^ = 5.31, n.s.) and their exclusion did not affect balancing. Full sample description and exclusion criteria are summarized in the supporting information (S1 Table). All procedures were approved by the Institutional Review Board at the University of Pittsburgh and in accordance with the principles expressed in the Declaration of Helsinki. Prior to testing, written informed consent was obtained from adults and participant’s assent and parent’s consent for minors. Participants and accompanying legal guardians were monetarily compensated for their participation.

### Task

**Fig 1:**
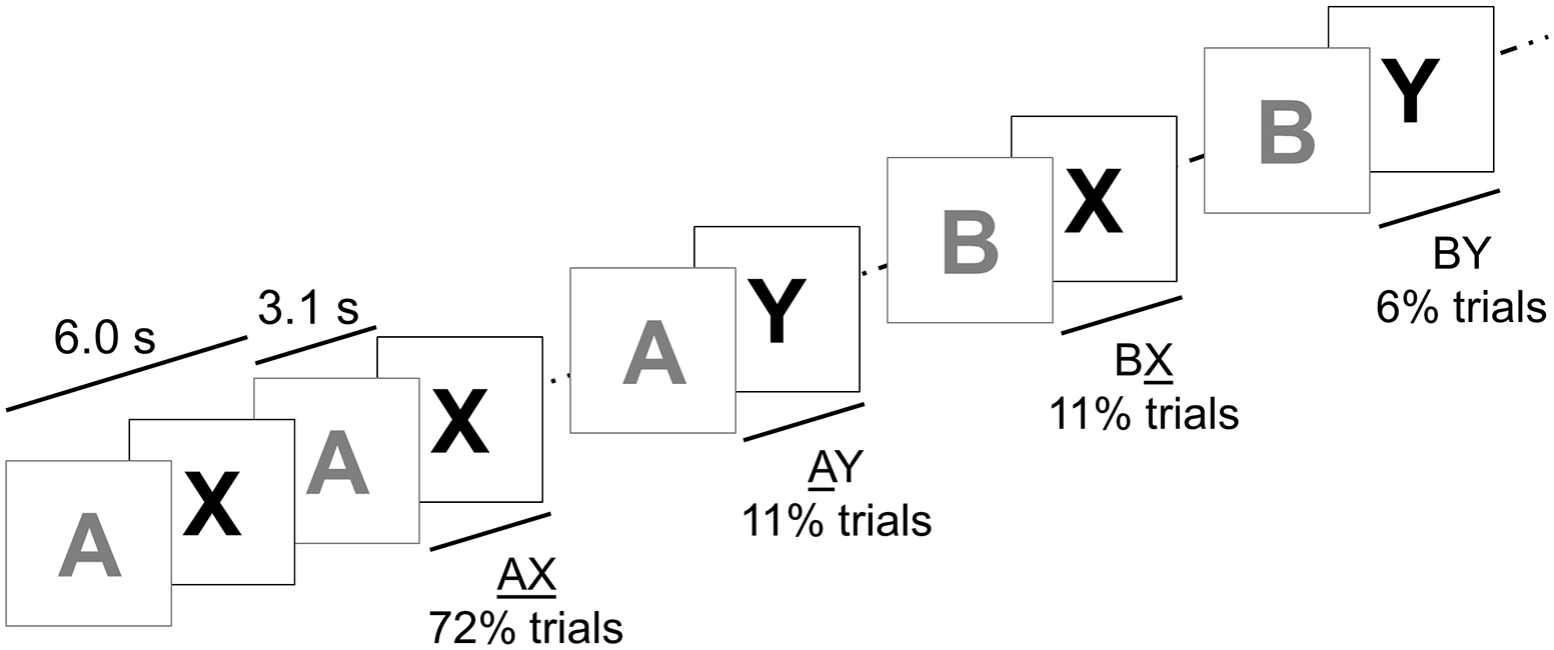
The expectancy AX-CPT paradigm. After training, 144 cue-probe pairs divided into 4 blocks were presented. Key-press responses—left (Target) and right (Nontarget) finger button press—were collected up to 1.5 s after cue onset and up to 1.3 s after probe onset. Acoustic feedback indicated correct (Target response on AX probes, Nontarget otherwise), incorrect or non-response. We employed the standard presentation provided by CNTRICS for E-prime [5,14]. Stimuli were Helvetica uppercase white (cues) or blue (probes) letters displayed on black background. Timings: Cue-probe onset interval: 3.1 s; cue-cue onset interval: 6.0 s; cue duration: 1.0 s; probe duration: 0.5 s.

Participants were presented with a series of 144 cue-probe letter pairs, and asked to respond as *Target* only when presented with an X-probe following an A-cue (“AX” trials, 104 pairs) and *Nontarget* otherwise, i.e. non-X probes following A (“AY” trials, 16 pairs) and for X probes following non-A cues (BX trials, 16 pairs) or non-X probes following non-A cues (BY trials, 8 pairs; Fig 1). Subjects were asked to respond as quickly and accurately as possible.

Since the expectancy adaptation [2] of the original AXCPT [15], the task has had several variants. The frequency of trial types and cue-probe delay durations have been varied in attempts to modulate CP. We tailored such parameters to the current age range of interest with the aim of deriving robust estimates while retaining task properties and comparability. We increased the relative proportion of critical AY and BX conditions over BY trials, which maintained the desired bias driving trial type frequency. As in other developmental studies we used a single cue-probe delay (3.1 s), an intermediate value compared to the ‘short’ (0–1 s) and ‘long’ (5–6 s) delays that are employed in some studies with adults.

### Analysis and measures

We will describe raw performance trajectories in terms of error rates and reaction times (*measures of performance*) and plot trajectories of control through standard indices (*model-based measures*).

In the framework of CP theory, the basic assumption is that the high proportion of AX trials produces a bias for Target responses that extends to trials sharing the cue or probe with AX. Accordingly, the use of context information can be more specifically indexed by the correct detection of AX as Target over and above the probe-driven bias that leads to false alarms in BX trials. This is operationalized by the signal detection measure d’-context [2]. The reliance on a cue-driven, proactive strategy can effectively reduce false alarms in BX trials, but also enhances cue-related chances of errors in AY trials. Thus the same proactive strategy would give rise to opposite effects in AY vs. BX trials and it would be effectively indexed by performance differences in these trials (AY-BX, [4]). AY and BX trials have also been contrasted to AX in terms of reaction times, producing cue-related and probe-related Interference measures, with the possible benefit of controlling for general reaction time differences [16]. Other approaches have employed shared variance across trial types rather than differences in mean values [9].

The same assumption underlies these prior approaches: the difference in performance across trial types is driven by their similarity to the Target. We will challenge this assumption by exploring data-driven relations in individual responses through principal component analysis (PCA, *data-driven relations across trial types*). By addressing the underlying structure of performance and its consistency across age, PCA also addressed the psychometric properties and the robustness of model assumptions.

The comparison across nontarget trials would also identify alternative strategy underlying AX responses. Indeed, a true discrimination of AX should be differentiated from a general response bias. To this end, BY trials, which share neither the cue nor the probe with the Target AX trials, are usually interpreted as a control condition and should lead to fast and accurate responses. Conversely, poor performance in BY trials could index generalized deficit confounds such as difficulties in suppressing a generic response bias or in following experimental rules. These internal controls are one of the strengths of the AXCPT as a specific CP test [3,5] and can be particularly useful in developmental studies which may be inherently prone to such confounds. However, this reasoning becomes circular if model-based assumptions are not met. Relations captured by data-driven relations such as PCA have been suggested to be more useful than other approaches in addressing sources of variability unrelated to the process of interest [17].

Finally, we also addressed the *chronometry of responses*. Here, we outlined the consistency of the parametric description of reaction times on accurate trials, their relationship to error responses, and provided a confirmatory and unified perspective on performance differences through hazard and speed-response functions.

#### Measures of performance

Error rate (ER) and reaction time (RT) were investigated using Generalized Estimating Equations (GEE [18]) on trial per trial observations with Age Bin and Trial Type as predictors. Errors were modeled as binomially distributed (logistic regression, logit link function) and RTs as normally distributed after a symmetrizing transformation (see *Chronometry of responses*; linear regression, identity link function). Coefficient estimates (exp(b), error odds ratios for logistic regression [19]) were used to compare the effects specific to each level controlling for other terms in the model.

Beyond age-related changes in mean performance (i.e. best fit of values predicted by Age Bin), Trial Type main effects provide a useful template to describe the sources of Age Bin*Trial Type interaction. These critical age specific deviations were investigated both in terms of trajectory (fitting of residuals after removal of Age Bin and Trial Type main effects) and within age bin pairwise contrasts. Bonferroni correction was applied to correct for multiple comparisons (0.01 alpha value).

#### Model-based measures

Indices based on individual accuracy were derived aggregating performance in AX and BX trials for d’-context (d’ = z[1-ER AX]−z [ER BX], [20]), and in AY and BX trials for the Proactive Index (PI = AY ER − BX ER or normalizing for total error rates differences ([AY-BX]/[AY+BX], [4]). For variance stabilization the arcsin transformation of squared ER was used. Indices based on parametric analysis of RT were computed on mean values after symmetrizing transformations of raw times. Indices contrasting cue-driven interference, AY-AX RT, vs. probe-driven interference, BX-AX RT [16], and RT based Proactive Indices were also examined.

#### Data-driven relations across trial types

We will refer to as *developmental-PCA*, the analysis performed *across* age bins and as *Age bin-PCA*, the *within* bin analysis. In addition, to capture patterns across broader stages of development, we performed analyses collapsing across subsets of bins, e.g. developmental-PCA1-2 refers to PCA run on participants from Age Bins 1 and 2 together. In order to compare values covering different variability ranges, PCA was performed on the correlation matrix of individual performance mean values, i.e. on normalized observations. Therefore, derived components are not unduly biased by differences in absolute values and effectively capture maturation of performance in terms of uncorrelated data-driven relationships.

As PCA approaches can be sensitive to outliers, we employed two strategies for outlier removal, with convergent findings. Aside the used standard deviation (SD) threshold, we explored more stringent values (down to 2.5 SD) and also the Hotelling’s T^2^ test to define outliers in terms of their multivariate distance from the center of the data set. The two approaches produced consistent findings with structure of loadings and scores for components explaining over 80% being unaffected by outliers removal. Since components accounting for less than 20% of the variability where generally less robust, we report the general pattern of the variance unexplained by main components in terms of residuals.

#### Chronometry of responses

Making mechanistic inferences from RTs depends on the relationship between RTs and accuracy. Moreover, the validity of parametric approaches relies on the actual shape of RT distributions. We investigated whether performance patterns could be accounted for by different temporal relations between RTs and errors both by parametric strategies and non-parametric approaches. The latter involve hazard and speed-response functions, which also provided a visually informative tool for inferences on the relation between RTs and accuracy [21].

The observed distributions for accurate RTs, in all trial types and for all age bins, was highly skewed and heteroscedastic, with variance being related to mean values. These properties were best captured by gamma distribution after introduction of a 200ms threshold parameter, which yielded strikingly better maximum likelihood estimation values over Gaussian and ex-Gaussian fitting [22,23]. Accordingly, a cube-root transformation on threshold subtracted values allowed the legitimate use of central-value approaches [24].

Given the overall relatively high accuracy, error trial RTs were not suited to addressing differences across age bins. We managed to show a distinctive, trial type specific pattern by clustering participants into three, wider age bins: children (8–12), adolescent (13–17) and adults (18–22 year-olds). This clustering is commonly exploited in developmental studies [25], was justified by the performance trajectories and it preserved the balance for demographic variables. These observations were substantiated by a non-parametric description of RTs.

Speed-response functions are the ratio of the empirical probability density estimates for Target over Nontarget responses and provide an estimate of the instantaneous relative probability of Target responses, i.e. they capture bias changes over time. Since they are derived from the same cumulative distribution—namely the distribution of all responses—we were able to derive the related hazard function: the conditional instantaneous probability of responding given that a response has not occurred yet. This approach allowed characterizing the quality of information processing beyond RT differences.

## Results

### Measures of performance

**Fig 2:**
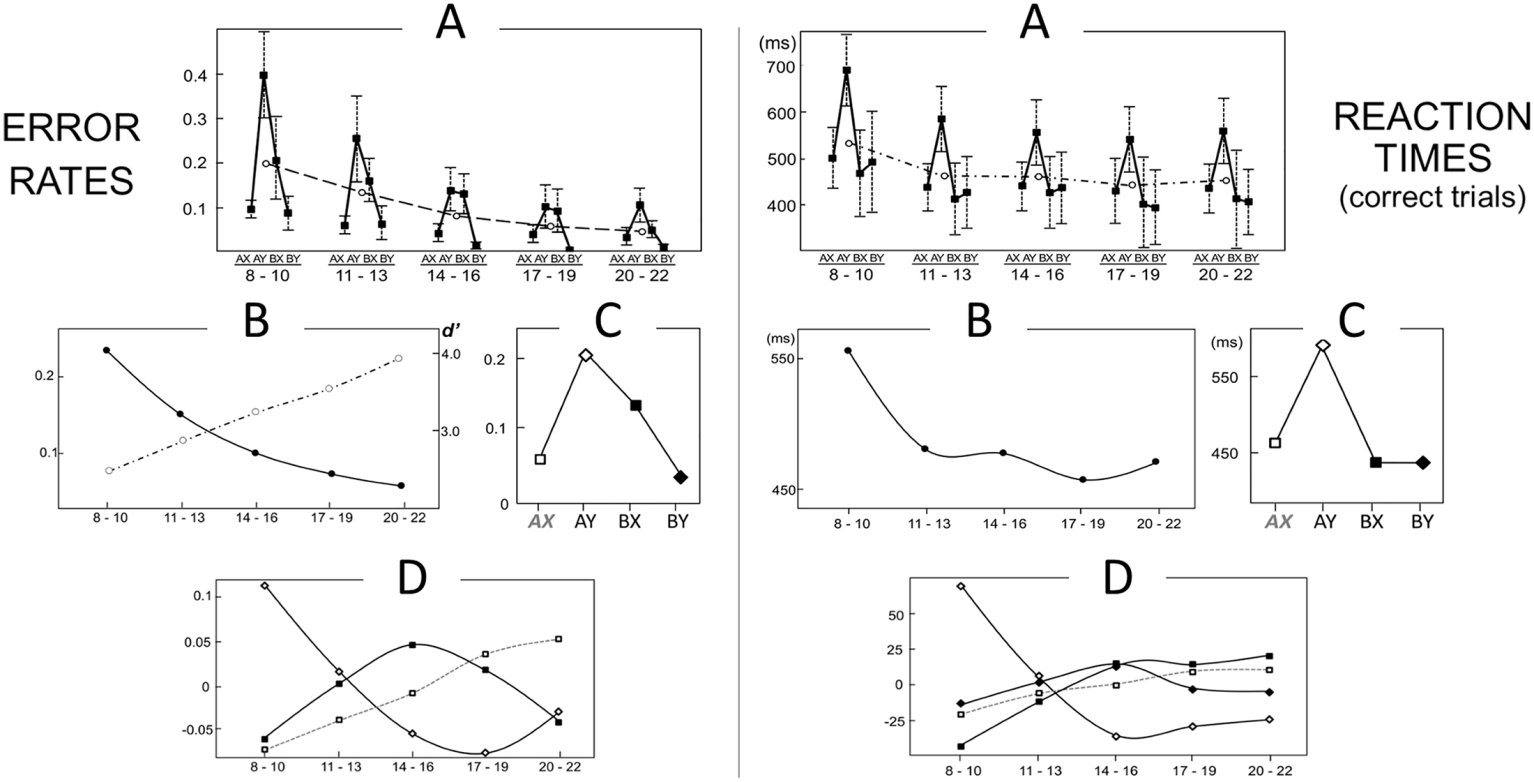
Performance measures: Summary (A) and regression analysis (B, C, D). In A, error rates and reaction times are displayed. Age Bin-wise averages for each trial type (filled squares) and across all conditions (open circles) show global monotonic improvements (dotted lines) together with clear differences across trial types. Respective marginal means show the main effects of age bin (B) and trial type (C); residuals are plotted in D. Accuracy generally improved with age across all trial types, while AY and BX appear to be stably more difficult than AX and BY. The pattern of residuals indicates that central Age Bins (14–19 years old) made relatively more BX errors. Similarly, youngest (8–13 years old) and oldest subjects (19–22) made more errors in AY. d’-context is also overlaid (open circles) on A, showing monotonic improvement. Reaction times were slower for younger subjects, with AY slower throughout the whole sample. Both effects were particularly marked in youngest subjects (8–13 years old). Such patterns suggest complex changes that involve differences both between and within identically cued trials.

Raw performance measures are plotted in Fig 2 and tabulated in S2 Table. For both ERs and RTs, the GEE showed highly significant main effects of Age Bin (χ^2^ = 674, p < .001; χ^2^ = 1081, p < .001) and Trial Type (χ^2^ = 551, p < .001; χ^2^ = 37, p < .001), and Age Bin*Trial Type interaction (χ^2^ = 28; p < .001; χ^2^ = 63; p < .001). Responses as a whole got more accurate and faster with age, with a steeper improvement for younger participants as underscored by a trajectory best captured by quadratic terms (R^2^ = .6, p < .001; R^2^ = .9, p < .001) and significant parameter estimates in Age Bin 1 and 2 for ER (3.6, p < .001; 2.1; p<0.001) and in Age Bin 1 for RT (1.6, p < .001).

For ERs, there was a main effect of Trial Type (p < .001, Fig 2C), AY and BX were consistently more difficult than AX trials, with error odds being 4.1 and 1.6 times higher respectively (p < .001; p = .02). Parameter estimates showed that BX errors were more likely in the intermediate age bins (Age Bin 2: exp(b) = 1.9, p = .025; Age Bin 3: exp(b) = 2.3, p = .002). Pairwise comparisons showed that within each age bin ER differences were in line with Trial Type main effects with the exception of lack of difference between AY and BX trials in Age Bins 3 and 4. This is in line with parameter estimates findings in showing a relatively greater difficulty of BX over AY in this age range. BY was confirmed to be the easiest trial type, showing virtually perfect performance from Age Bin 3 onwards. However, in Age Bin 1 and 2 BY accuracies did not differ from AX. After main effects removal, residual variability in AY and BX trials was consistently best modeled by linear and quadratic terms with fairly overlapping magnitude but opposite direction (k—.30(Age Bin) + .25(Age Bin)^2^+err, p = .002, p = .013; k+0.30(Age Bin) -.29 (Age Bin)^2^+err, p = .003; p = .004; Fig 2D).

For RTs, a main effect of Trial Type was supported by all pairwise contrasts except BX-BY (Fig 2C). Correct responses in BX and BY appeared to be equally faster (exp(b) = 0.7, p < .001; exp(b) = 0.7, p < .001) than in AX trials, while responses in AY appeared to be overall much slower (exp(b) = 2.2, p < .001), particularly for the youngest participants ([Trial Type = AY]*[Age Bin = 1], exp(b) = 1.3, p < .001). Pairwise comparisons within each age bin showed the same significant differences predicted by Trial Type main effects, with the only notable exception being lack of difference between AX and BY trials in Age Bin 3. This observation seems to be related to a complex pattern of residuals (Fig 2D), with Age Bin specific relative differences that could not be captured by low order trajectories.

### Model-based measures

We observed striking differences in the trajectories of d’- context and Proactive Index. D’- context improved linearly from 2.40 to 3.75 (F_170_ = 14, p < .001; d’ = 2.2+0.33(Age Bin), R^2^ = .3, p < .001, overlaid in Fig 2B). Proactive Index showed a quadratic relation with age (F_170_ = 7.7, p = .001; AY-BX = 0.5–0.2(Age Bin)+.05(Age Bin)^2^, p < .001), consistent with the observed higher ER in AY in youngest and oldest participants and lack of difference in AY and BX ER in Age Bin 3 and 4 (highlighted in Fig 1). Such difference was emphasized by normalization of AY-BX, which equated Age Bins 1 and 5 while retained the Age Bin 1 to Age Bin 3 difference (p = .03). Accordingly, only the estimation of a quadratic polynomial contrast was significant (R^2^ = 0.42, p = .02; linear: R^2^ = 0.19, p = .87).

RT based measures reiterated results from pairwise contrasts. For indices of interference, no significant BX-AX differences (F_4,170_ = 0.4, p = .79) but age dependent AY-AX differences (larger for Age Bin 1–2 and smaller for Age Bin 3–5, F_4,170_ = 5.1, p < .001) were observed. The RT-based Proactive Index AY-BX strikingly differs from the ER based, not showing differences across ages (F_4,170_ = 1.0, p = .40).

### Data-driven relations across trial types

**Fig 3:**
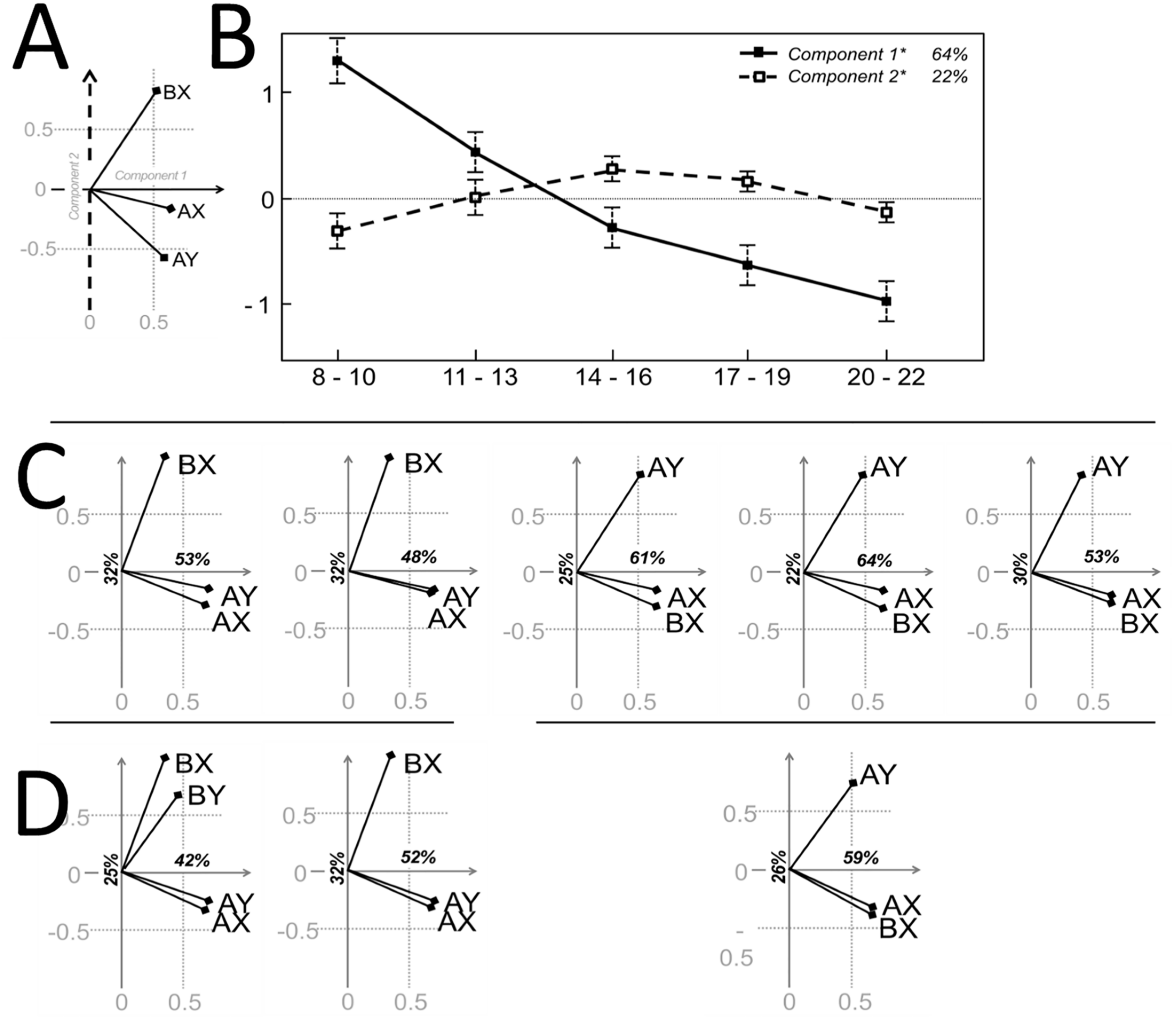
Principal components analysis: Error rates. The variables contributing to the first two components (i.e. loadings biplot, A) and representation of the observations in the principal component space (i.e. scores distribution, B) for the developmental-PCA on AX, AY and BX error rates are consistent with findings from the regression analysis: the main directions of variance track improvement across all conditions and the separation of AY and BX performance that contrasts central age bins with younger and older subjects. However, loadings biplots for Age Bin-PCAs (C) show different relations across Trial Type accuracies for bins 1 and 2 vs. bins 3, 4 and 5, which remain relatively stable across the respective age ranges (Developmental-PCAs 1–2 and 3-4-5, D). While performance in older subjects in AX and BX trials shares consistent variance, cue-related differences dominate performance in children as BY variability in this age range is largely shared by BX.

**Fig 4:**
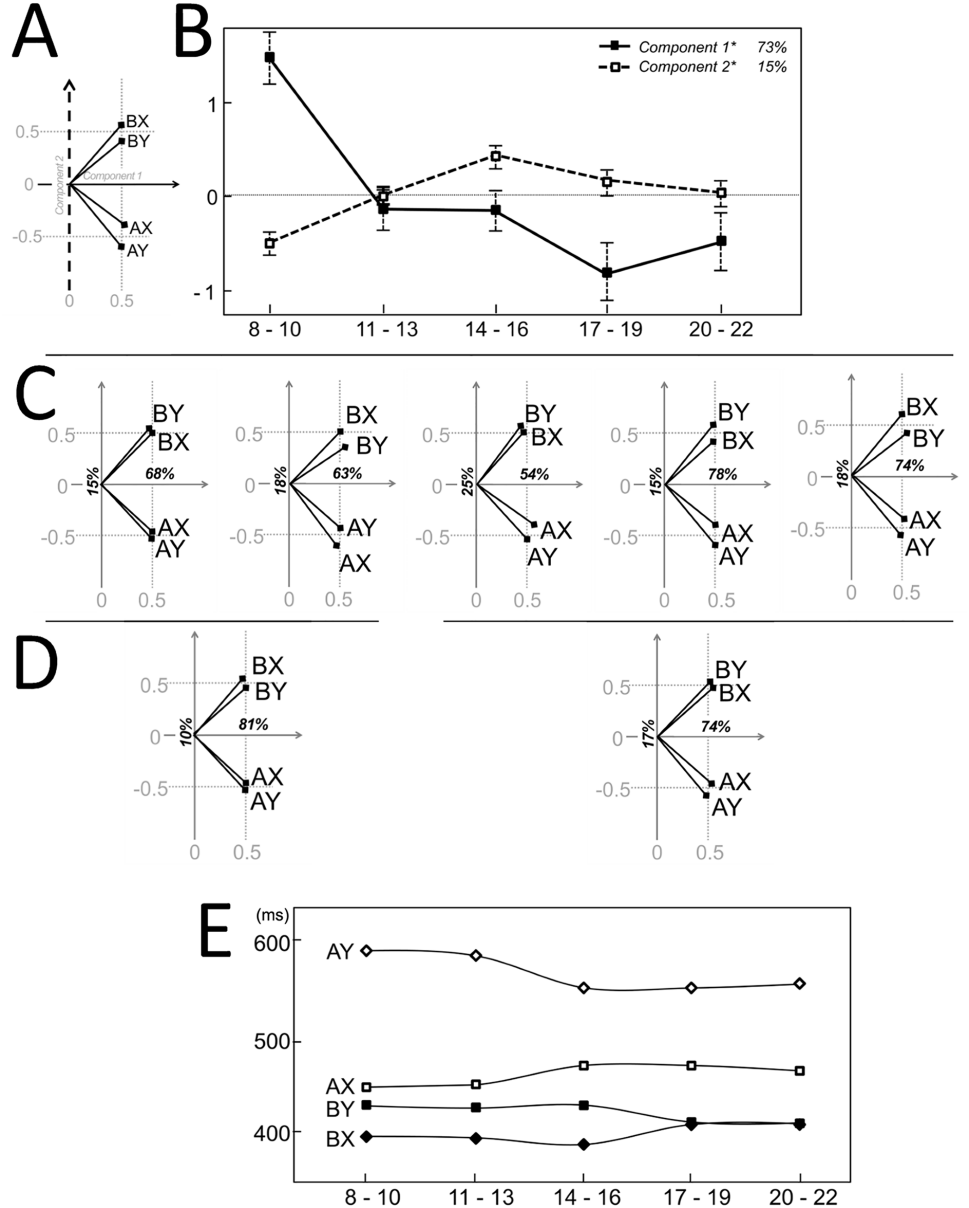
Principle components analysis: Reaction times. Following the format of Fig 3, displayed are the loadings biplot (A) and scores trajectories (B) for the first two components of developmental-PCA on mean reaction times on accurate responses; consistent loadings biplots for Age Bin-PCAs (C) and Developmental-PCAs 1–2 and 3-4-5 (D). Over 80% of the variance both across and within age bins is accounted for high correlation across all trial types and cue-related differences. Residual variability highlights slower responses for youngest subjects on Y probes (E).

Developmental-PCA across all Age Bins, both on ER and RT, revealed a high degree of correlation in the performance across trial types. The first component (C1), explained 64% of the ER variance and 73% of the RT variance. All trial types showed high and comparable loadings, indicating that C1 captures average performance across all the trial types (Figs 3A and 4A). Accordingly, scores parallel age main effects observed in regression analysis (Figs 3B and 4B): ER C1 decreases fairly linearly (F_4,170_ = 22, p < .001; linear fit, R^2^ = 0.35, p < .001) while RT C1 plateaus after a steep decrease (F_4,170_ = 11, p < .001; inverse fit, R^2^ = 0.21, p < .001). Secondary components (ER C2 and C3, RT C2, C3 and C4) captured relations between specific trial types. We observed age-dependent differences in the distribution of individual scores paralleled by different data-driven relations in Age Bin-PCA.

For error rates, C2 explained 22% of the variance, with AY and BX having high and opposite loadings (AY 0.59, BX 0.57). Scores were distributed differently across age bins (F_4,170_ = 5, p < .05) and, as predictable from AY-BX differences, traced an inverted-U shaped trajectory peaking at Age Bin 3. However, Age Bin-PCA showed two distinct structures for Age Bins 1, 2 vs. Age Bins 3, 4 and 5. Within each Age Bin most of the variance was not shared across all trial types, with one trial type relatively orthogonal to the other two, BX for Age Bins 1–2 and AY for Age Bins 3-4-5. Accordingly, for Age Bins 1–2, C1 and C2 separated AY-AX from BX; for Age Bins 3-4-5 AX and BX were highly correlated along C1, while AY became progressively less correlated (decreasing AY loading on C1 (0.4–0.4–0.3), and AX and BX loadings on C2). Developmental-PCA1-2 and Developmental-PCA3-4-5 showed consistency with the respective component Age Bin-PCAs (Fig 3C and 3D). The inclusion of BY ER in both developmental and binwise PCAs for Age Bins 1–2, where no ceiling effects in BY accuracy were observed, highlighted the alignment of BY to BX ER, i.e. loadings on C2 over 0.5 in all PCAs. The relative increase in BX ER observed from Age Bins 1 to 3 seems therefore to be related to differences in accuracy shared by both B-cued trials. The residual 12% related to AY-AX differences (ER C3, AX -0.7, AY 0.56), with scores that did not appear to differ significantly across age. However, PCAs for Age Bin 3-4-5 showed that the distinctive aspect of accuracy of older participants relates to a distinct performance of AY that was increasingly from the high variance shared by AX and BX.

Reaction time C2 explained 15% of the variance and defined an orthogonal direction that separated the AX-AY and BX-BY pairs. Scores increased from Age Bins 1 to 3 and then decreased to intermediate values for Age Bins 4 and 5. This general structure was substantially maintained within each Age Bin (Fig 4C): for all Age Bin-PCAs most of the variance was explained by the correlation across all trial types and the orthogonal separation of A-cued and B-cued trials, with absolute values of loadings on C2 never falling below 0.4 for all trial types in all PCAs. Age Bin-PCA 2 and 4 appeared to be more vulnerable to outliers’ removal and the described structure emerges only after exclusion of the most distant observations (~10%). However identical correlations along C1 and C2 were also observed for developmental-PCA 1–2 and 3–5 (Fig 4D), with cue-related differences in RT representing an important aspect of performance at any age. C3-C4 separates AY-AX and BY-BX. The reconstruction of the RT pattern based on C3-C4 components, i.e. the residuals after C1-2 removal (Fig 4E), shows similar differences for AY-AX and BY-BX in younger bins, with responses in Y probe trials slower than the respective, equally cued X trials: the AY-AX difference disappears from Age Bin 3 onwards, replicating evidence from cue-related interference index, while the BY-BX difference disappears later, from Age Bin 4 onwards, in line with overlapping BY-AX RTs for Age Bin 3 (Age Bins 1 to 5, for AY-BX, p = .02, 0.03, 0.58, 0.62, 0.12, respectively; BY-BX, p = .01, 0.03, 0.01, 0.55, 0.63, respectively).

### Chronometry of responses

**Fig 5:**
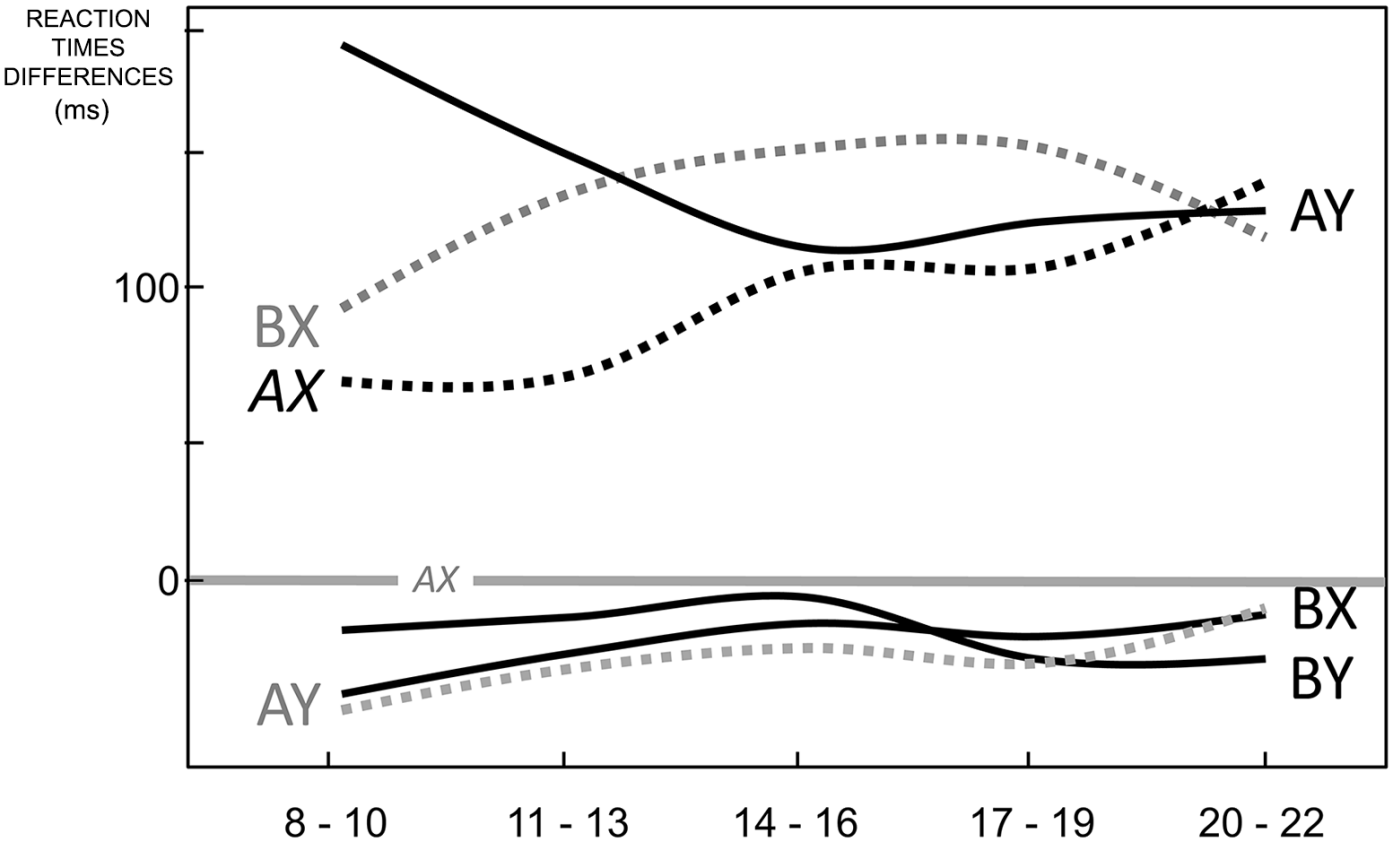
Chronometry of responses: Mean RT for both correct and incorrect responses. In order to highlight relative differences the RT on AX correct, Target responses is subtracted from all RTs. Findings from analysis on accurate trials (solid lines) are readily visualized and paired with related incorrect responses (dotted lines). The stable differences in RTs suggest that most responses in incorrect trials arise from chronometrically distinct processes, slower in AX and BX trials, and faster in AY.

An overview of mean reaction times for both correct and incorrect responses is provided by Fig 5. For all trial types, at all ages RT on error trials were very different from correct responses. GEE analysis on all RT, either using accuracy or response type as covariates, showed both main effects and significant interactions with Trial Type (χ^2^ = 548; p < .001; χ^2^ = 394; p < .001), but no evidence of three-way interactions (χ^2^ = 4; p = .86; χ^2^ = 10; p = .34). Direct pairwise contrasts confirmed clear separation of the two distributions for each trial type in all age bins. In A-cued trials, regardless of accuracy, Target responses were faster than Nontarget responses. AY Target, incorrect responses were faster than AX correct responses in all Age Bins except 4 and 5. AX Nontarget, incorrect responses were as slow as correct AY trials, in all Age Bins except 1 and 2. In BX trials, the reverse pattern was observed: Target, incorrect responses were slow, substantially overlapping with AX Nontarget responses aside from a tendency of being slower for central age bins that becomes significant at Age Bin 3.

**Fig 6:**
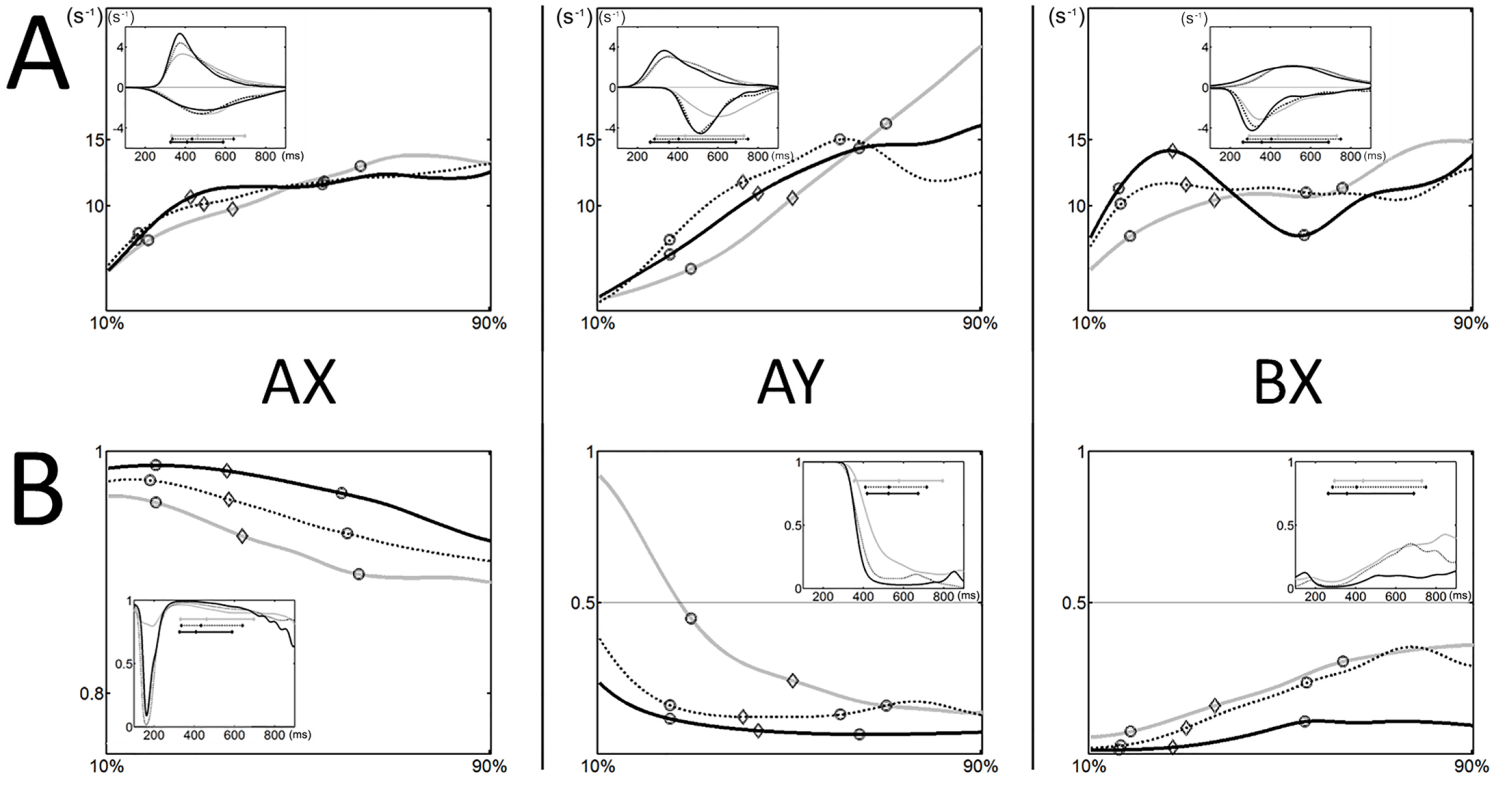
Chronometry of responses: Non parametric description of reaction times. Hazard (A) and speed-response (B) functions responses in AX, AY and BX trials are plotted for children (8–12 years old; gray line), adolescents (13–17; dotted line) and adults (18–22; black line). We display the central 80% of responses and curves are translated so that curves can be compared whenever they occurred in absolute time. The pattern of the remaining 20% of responses—the fastest 10% and slowest 10%–is described in the text. Markers on each line correspond to 25%, 50% and 75% of responses and help to verify such alignment. Insets show the complete curves and localize the subset of responses on absolute time scale. The hazard function insets contrast the empirical probability distribution functions for Target responses (positive direction) vs. Nontarget responses (for clarity, plotted in negative direction). Note the skewed profile that motivated the employed transformations for parametric analysis. Hazard functions on BX trials suggest a progression towards more prompt responding with age. Speed-response functions represent the instantaneous probability of Target response. Identical response biases at probe-onset and subsequent qualitatively similar information processing are evident for AX trials. For children, responses in AY trials are distinguishable for a protracted indecision period. Adults appear to maintain a clear Nontarget response bias throughout the whole response window as evident for both AY and BX trials.

Non-parametric approaches extends these observations by addressing response time and accuracy jointly. In Fig 6 speed-response functions estimate the instantaneous relative probability of a target response. Curves are plotted for the central 80% responses, i.e. not including the fastest 10% and slowest 10% responses. Profiles show a trend consistent with a deviation from an initial bias for all trial types at all ages. While AX curves displayed fairly comparable probe-processing across all ages, the AY curves are distinctive for youngest participants and BX curves for oldest participants. In AY trials, following the first 10% of responses, the relative probability of a target response for the youngest participants is strikingly high and decays slowly going through a substantial response indecision period. On the other hand, older participants show clear evidence of a prompt probe-driven suppression accompanied by reorientation towards a Nontarget response, as indicated by a monotonic hazard function. In BX trials, oldest participants also showed a reduced slope of the probe-driven increase of relative frequency of Target responses, accompanied by prompter responding as indexed by an early peaking in the hazard function. Of note, the frequency of errors in the fastest 10% and slowest 10% responses were in agreement with overall ER differences with one exception: youngest participants had a lower frequency of Nontarget responses in the fastest AX responses.

## Discussion

### Standard interpretation of results

In the current study we provide evidence of a protracted and complex refinement of AXCPT performance in participants aged 8 to 22 years old. The task aims at capturing CP by operationalizing it as the context appropriate detection of the probe X in AX, Target trials. Other trials share variable similarity to AX and they can be exploited both to rule out nonspecific differences (i.e. generalized improvement, changes in response bias) and to derive standard indices that are commonly used to capture specific cognitive control profiles. We tracked maturation through such standard, theory-driven indices and also investigated their validity by expanding behavioral analysis to include correlation and chronometry of responses.

**Fig 7:**
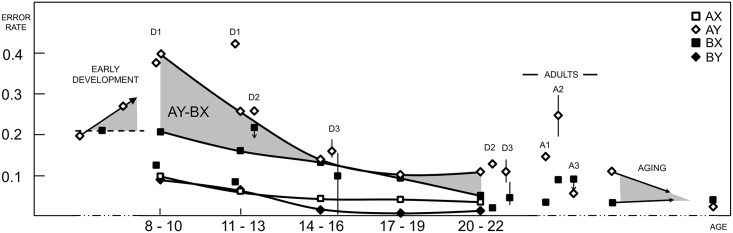
AY-BX error rates in the AXCPT across the life-span. Plotted are raw error rates formatted to highlight AY and BX trajectories (connected markers) and their difference (shaded in grey). The latter has been used in prior literature to index context processing or proactive strategies of control. Overlaid (unconnected markers) are AY and BX error rates reported in all prior AXCPT studies over development (D 1–3) and in a selection of studies on adults that employed similar parameters (A 1–3). When more than a single cue-probe delay was tested, the range is reported and the marker is positioned on the interpolated value corresponding to what used in the current study (3.1s). Early development and aging data were adapted for display as protocol differences do not allow a direct comparison: the depicted trend reflects the qualitative interpretation provided by authors: Chatham et al. suggest an increase of proactive strategies from age 3.5 to age 8 [9]; Braver et al. hint to an inversion of this trend with aging, with negative AY-BX difference in the elderly [26]. Despite task parameter and sample differences, higher AY-BX differences at the two extremes of the explored age-range appears to be broadly consistent with AY-BX differences reported in other developmental studies and with the extrapolation of the trajectories in adult studies. We provide evidence indicating that this apparent similar reliance on proactive strategies is accompanied by critical differences in their control content. Of note, most of the cited studies rely on heterogeneous analytic strategies with variable reliance on accuracy and reaction times. Still, conclusions are generally driven by the same model-based reasoning and inconsistencies across measures are explained in terms of sensitivity differences. Furthermore, AY-BX predictions have been challenged by a minority of studies in young adults too. Here we exploit the case of AY-BX error rates as a proxy for standard accounts of context theory emphasizing the need to investigate findings beyond model-based accounts, especially in populations with complex cognitive profiles. (D 1–3: [10–12]; A 1–3: [16,27,28].

Since the patterns across trial types varied across ages, the observed maturational changes can be explained neither in terms of a generalized improvement nor by a change in response bias. By comparing AX accuracy with BX Nontarget trials, we derived d’context, a standard CP index [2] and showed an improvement at a constant rate over the observed age range. Dual mechanisms theory parsimoniously interprets underlying strategies along a unique proactive-reactive dimension and postulates that an increase in proactivity would have opposite effects in these two trial types. Accordingly, Proactivity is generally indexed by comparing performance in AY and BX trials (AY-BX, [4]). This AY-BX index of proactivity showed a non-monotonic trend, with a decrease from childhood to adolescence followed by an increase from adolescence to adulthood. Fig 7 summarizes ER findings from this study, highlighting this primary AY-BX finding. The figure also overlays the results from previous AXCPT studies in healthy participants. This compiled summary includes findings spanning both within and outside our age-range in order to underscore the significance of our findings in completing a putative normative trajectory through the whole life-span. While our study suggests somewhat counterintuitively that children and adults are equally proactive, results are in general agreement with previous sparse observations in smaller samples from within the same age range. However, further analyses clarified this misleading apparent equivalence in children and adult performance by capturing critical differences in the pattern of relations across trial types and chronometric profiles across these age ranges.

### Further analyses: Age related differences in proactive strategies

While children and adults have overlapping AY-BX ER, their performance differs in other trial types. Specifically, they showed high vs. low BY ER and high vs. low AY-AX RT interference, respectively (Fig 2). In fact, a direct interpretation of the RT findings could provide a sharply different perspective. For instance, a RT based AY-BX index does not differentiate age bins and clearly contrasts with ER findings. These observations suggest that the standard univariate analyses could ignore important sources of variability, not predicted by theory and better revealed by data-driven approaches. Moreover, a consistent account should reconcile ER and RT observations into a unified account, examining the possibility that responses at different ages arise from chronometrically different processes or from different relations between speed and accuracy.

Beyond the general positive correlations across trials, the data-driven exploration of ER showed that second main source of variability across the whole age-range was related to a modest anti-correlation between BX and AY trials (Fig 3). This would be in line with the dual mechanisms theory predictions. However, analysis within age bins showed that such result could have been biased by unbalanced contribution of different patterns. In fact, some degree of AY-BX anti-correlation is observed only in Bin 3, 4 and 5. A different pattern was observed in younger participants, where the second main source of variability is captured by cue-related differences with A-cued trials being more positively correlated with each other and negatively correlated to B-cued trials. Importantly, it was possible to make this observation only in children as BY ER was not at ceiling. This further emphasizes the limits of the analysis performed across the whole age-range.

The data-driven RT analysis highlighted that cue-related differences in RT represents an important aspect of performance at any age (Fig 4). Over 80% of the variance both across and within age bins is accounted by high correlation across all trial types and cue-related differences, again with A-cued trials being more positively correlated with each other and negatively correlated to B-cued trials. The trend of this pattern tracked through such second component scores shows that the balance is shifted towards relatively faster B-cued trials in children and adults and towards faster A-cued trials in adolescence. Analysis of residuals suggested slower responses for youngest subjects on Y probes, in both A-cued and B-cued trials. Such uncorrelated age-specific Y-probe slowing is in agreement with univariate RT analysis findings—slower AY responses in children and BY responses in adolescence. Note that the above mentioned sources of variability would hinder the detection of trial specific, univariate differences.

Our extension of RT analysis to incorrect trials revealed complementary insights (Fig 5), with both a general pattern across the whole sample as well as age-specific differences. At all ages, incorrect responses RTs were stably different from corresponding correct responses, being slower in AX and BX trials, and faster in AY. As a consequence, the fastest responses were A-cued Target and B-cued Nontarget, regardless of accuracy. In other words, correct and incorrect responses arise from chronometrically distinct processes that are mainly cue-driven and accordingly can be seen as preparatory to responses on probes. Since such a pool of fastest responses occurs in a short time frame after probe onset—just as fast as or even faster than the most frequent AX, Target responses—it is likely that performance at all ages is affected by cue-driven preparatory mechanisms involving some degree of early response selection.

However, since both B-cued Nontarget responses were faster than AX Target responses, it can be inferred that the two cues also trigger different levels of probe processing, with a greater processing and related chronometric cost in A-cued trials. Of note, such observations are congruent with a proactive strategy involving an early response selection driven by both cues—A-Target and B-Nontarget—and probe processing triggered only by A-cues. This strategy would optimize AXCPT performance by allowing responding as fast and as accurately as possible. However, the perspective from incorrect trials suggests age-related differences that adds to findings from correct responses in suggesting different access to such strategy.

In A-cued trials, we emphasized that successful Y-probe processing comes with a further delay across all ages but this appeared to be more taxing in youngest participants. In children, Target responses were faster in AY trials and Nontarget responses were faster in AX trials, while from adolescence onwards Target and Nontarget responses had overlapping RTs in each trial type. In fact, children showed relatively faster responses in error trials than in corresponding correct responses, while in older participants response type accounted for RTs differences regardless of accuracy. Such evidence suggests that in children, while some level of probe processing is clearly engaged, this process is faster when it fails to map onto the right response. Conversely, in older participants each probe leads to different operations which are more taxing in AY trials but in both cases they affect the likelihood of selecting a certain response without affecting the time of its execution.

B-cued trials do not allow disentangling accuracy from response kind as all Target responses are incorrect. They also provide only a partial account of probe specific processing as the small number of incorrect BY responses do not allow their inclusion in the analysis. However, recall that the analysis from Correct, Nontarget responses suggested that children have a relative disadvantage in processing BY and adolescents in BX trials. While BX Target are slow at all ages, even such responses appear to be particularly slower in adolescence, suggesting that such error occurs on the ground of a slow process that is more represented in adolescence.

Of note, failure to establish appropriate cue-driven mechanisms can still contribute to a portion of correct responses through probe-driven (e.g. slowest AX, BX and BY) responses, or probe-insensitive (e.g. fastest AX) responses. However, the observed difference in mean RT for correct responses and errors suggests that most errors at all ages might be more closely related to failures to engage cue-driven mechanisms. While the standard AY-BX index suggests similarly higher proactivity in children and adults, RTs analysis suggests that the preparatory mechanisms are qualitatively different.

Such complex pattern of results suggests a provisional interpretation. Until mid-adolescence, performance is affected by sources of conflict different from the ones postulated by prior AXCPT models (e.g. differences in AY vs. BX performance, Fig 7). When youngest participants are asked to respond “Target” only on frequent AX-trials, the rare Nontarget trials appear to be approached with a cue-driven strategy that drives response selection for both cues. Such a strategy does not seem to extend to cue-specific probe processing, as children show a tendency to handle probes similarly regardless of the cue. This observation is more consistent with an ‘X-go vs. Y-stop’ mapping rather than the postulated Target-Nontarget mapping. Note that since the preselected response is correct for both AX and BX trials, on X probes this behavior would be reinforced for both cues. Crucially, in BX trials such strategy leads to Nontarget responses that largely bypasses the potential conflict expected for X-probe processing. As a result, the hypothesized double dissociation commonly postulated by proactive-reactive control shifts on AY and BX trials loses discriminative power.

A sharp change in performance patterns across trial-types is observed at age 14–16. Some degree of anti-correlation between AY and BX responses emerge, the earlier difference in performance in AY over AX trials is reduced and all B-cued responses relatively slow down. These changes suggests improved probe-related control with better mapping into the Target vs Nontarget dichotomy. While comparatively slower responses in BY trials suggest the persistence of rudimentary cue-driven mechanisms, these are reduced and thus do not permit bypassing the conflict related to X-probes: BY errors disappear but BX errors increases. Such a pattern is consistent with dual mechanisms account of a reactive strategy, as indexed by minimal AY-BX ER differences.

Against a backdrop of similar correlations, later developmental changes evince a continuous refinement of performance into early adulthood. Errors decreases in BX trials and accuracy in A-cued trials reaches a plateau. Such a pattern is line with an optimal proactive strategy that involves cue-specific preparatory mechanisms and differential probe-processing in line with Target vs Nontarget mapping rather than simple response selection. The existence of these different cue-driven strategies is a key impediment to a simple mapping of strategies along the same proactive-reactive dimension and helps to reconcile the otherwise puzzling findings employing standard indices. While such a proactive strategy underlies the observed high Proactivity (AY-BX) and high CP (d’context) in adults, a more rudimentary proactive strategy underlies high Proactivity (AY-BX) but low CP (d’context) in children. In fact, if CP is the context appropriate recognition of X-probes (Target vs NonTarget), a strategy that implies a weak representation of probes in terms of Target vs. Nontarget suggests poor CP per se. By this perspective, the emergence of Target vs. Nontarget mapping in adolescence allows us to understand how a low Proactivity, probe-driven strategy leads to intermediate CP and performance.

Results from non-parametric approaches appeared to agree with such an interpretation. They confirmed critical differences between proactive strategies and helped to reconcile the RT and ER findings. Hazard and speed-response functions characterize information processing and response bias after probe-onset by capturing the probability of responding and of such responses being the Target response, respectively. Profiles showed a general trend consistent with a deviation from an initial response bias for all trial types at all ages, but they also captured age and trial type specific patterns. AX curves suggested comparable probe-processing across all ages, while AY curves were distinctive for children and BX curves for adults (Fig 6).

Children showed a greater degree of Target response bias which appeared to be comparable across both A-cued trials. In AY trials this was accompanied by a probe-driven, delayed response adjustment. As a result, in this trial type children appeared to go through a substantial response indecision period—when both responses are equally probable—which is not observed in older participants who showed a prompt probe-driven suppression of the Target response. Adults, showed a prompter response in BX trials accompanied by a reduced slope of increase in the relative frequency of Target responses. As a consequence, the distinctive feature of adults’ curves is the maintenance of a clear Nontarget response bias throughout the whole response window for both AY and BX trials. Such evidence confirms the role of cue-driven mechanisms which are characterized by response selection and differential probe-processing and are differently accessed at different ages.

Of note, such chronometric analysis can be interpreted in terms of robust information processing differences beyond absolute RTs differences. This was achieved by aligning curves as to match age specific empirical distributions and focusing on central responses. We observed that the ERs corresponding to the fastest 10% and slowest 10% responses were in agreement with overall ERs with the exception of fastest AX responses in children that were significantly less likely to be incorrect. Indeed, in A-cued trials older participants are more likely to produce fast (occurring in the first ~300 ms) yet probe-specific (being absent in AY trials) responses. The benefit of probe-specific responses observed in more mature performance might bear the cost of a few trials with blurred cue-driven response selection. This can be potentially due to an increased probability of mistakenly engaging B-cue preparatory mechanisms or to A-cues not completely suppressing Nontarget response biases. Either way, this can be contrasted with the more robust response selection observed in children.

In conclusion, we showed that the standard assumptions underlying AXCPT performance are valid only from adolescence onwards. This age range was confirmed to be critical in performance development and appeared to be characterized by a reactive strategy that later matures in an optimal proactive strategy. Children access a different, suboptimal proactive strategy. Such a difference was obscured by the theory driven AY-BX indexing and violates the assumption of a unique reactive-proactive dimension. Such incongruence needs to be addressed examining the reasons underlying such qualitative differences, assessing the relation to other constructs and their developmental time courses. Indeed, the very raison d’etre of a control function is an integrative, optimal recruitment of multiple processes. Therefore, differences in cognitive control across development cannot be addressed without referring to subordinate or concurrent functions.

### Context processing and related cognitive functions

Working-memory maintenance functions are known to mature through late childhood, with more prolonged maturation observed when tasks involve manipulation or interference [29]. Throughout our sample, performance seems to be accompanied by early response selection, with suppression of the chronometric cost of X-probe processing in B-cue trials, i.e. all B-responses are equally faster than AX responses. Such a strategy can be effective when maintenance is challenged [26]. Children might exploit early response selection as a rudimental yet robust form of storage. Note that this could even take the form of actual, physical preparatory hand movements. On the other hand, we did not expect our task adaptation to be taxing on maintenance functions for adults. Yet we noted that early response selection is the optimal strategy in B-cued trials, if its implementation does not come with a cost in A-cued trials.

Inhibition plays an important role in the development of controlled behavior, and possibly in explaining the poor performance in AY trials observed in children. However, evidence suggesting modest behavioral improvements through late childhood in purely inhibitory tasks, i.e. Stop-signal and Go/No-Go paradigms [30], contrasts with the sharp early improvement and later stabilization of AY errors and reaction times. The delayed improvement in BX trials could depend on maturation of inhibition, which critically cannot explain the late relative worsening of AY responses. Context theory captures the late pattern by conceptualizing inhibition as emerging from top-down biases related to active goal maintenance [31]. Differences in purely inhibitory functions are ruled out by contrasting suppression of probe-driven vs. cue-driven response biases. Again, the observed loosened X-to-Target relation hinders this internal control in youngest participants. Improvement in AY trials in adolescence is more parsimoniously explained by a general reliance on probe-driven, reactive strategies. This shift can be effective only if maintenance and retrieval are adequate, and a further development of these functions might underlie the better accuracy on slow BX responses that partially differentiates adults. However, an improvement in maintaining symbolic cues is not sufficient in explaining cue-related differences as they importantly involve correlated changes in RTs across trials sharing the same cue at all the observed ages.

Cues contribute critically to the update of task relevant information postulated by CP theory. However in order to quickly and correctly bias probe-response associations cue representation needs to be procedurally relevant: the mapping and maintenance of stimuli in the Target-Nontarget dichotomy involves relations that are not about the ‘what’, e.g. B vs. A, but about the ‘how to’, e.g. prepare a Nontarget response vs. prepare a Target response *and* monitor probe identity. As a whole our results emphasize that the general definition of context cannot be operationalized without insights on how the challenge posed by “respond as quickly and as accurately as possible” is addressed and how such optimization problem is constrained by the specific limitations of developing systems. Conversely, if context is viewed as “the subset of representations within working memory that governs how other representations are used” [31], development of AXCPT performance can provide insights about the lower level representations that underlie mature CP and the relation of CP to constructs and paradigms related to such function.

Stimulus-response mapping is governed by rules or task-sets [32]. Development of AXCPT performance can be interpreted as maturation of cue-specific task-sets. As cue information can be exploited not only for selecting a response but to draw inferences about the probability of its inhibition, task-sets can be conceptualized as involving different associations between response selection and attentional shifts (probe identity vs. probe onset). There is evidence suggesting that suppression of a specific response can even occur as a preparatory mechanism [33]. Such proactive inhibition would reduce the reliance on purely probe-driven, reactive inhibition in a cue specific manner and task-sets would differ in its recruitment. Regardless of how each task-set is conceptualized it is critical to stress the importance to address the relation between such constructs. Depending on what associations are postulated at the task-switching vs. the CP level, either the two concepts are overlapping or task-switching is a lower level process. If the constructs are overlapping, task-switching reconfiguration is a sufficient operational definition of CP. On the other hand if task-switching is a subordinated function, its development is necessary to define CP operationally.

Task-switching consists of separable processes: task-set suppression, which matures by adolescence, and rule representation, which appears to undergo protracted development into early adulthood [34]. From this perspective children’s strategy can be held to compensate for immature task-set suppression capabilities. Failures to engage configurations prior to stimulus onset require the probe-driven reconfiguration that characterizes performance in adolescence. Our results are consistent with the conceptualization of task-set reconfiguration as a probabilistic event [32,35] and suggest that development might entail decreasing failures to engage cue-specific preparatory mechanisms. Note that in general implementing multiple task-sets is not a direct measure of the repertoire of rules represented. Separate task-sets can be advantageous but such advantages need to be weighed against task-switch costs. Thus, while the repertoire of represented rules has been proposed to expand with age, we suggest that through development task-switch cost can be a factor limiting performance. On the representation level, the so called nonspecific, mixing cost could contribute to the globally slower performance in children. Furthermore at this age procedural, specific costs can make the implementation of two rules particularly disadvantageous. Other constraints would limit the content of each task-set, its complexity and the preferred resources involved.

AXCPT performance should be interpreted in light of the flexibility in strategies allowed by the task itself. Such flexibility can be exploited by developing cognitive systems to adapt performance to their specific limitations. Dual mechanism accounts of control postulate specific constraints by measuring these adaptations: the reliance on probe-driven, reactive mechanisms is interpreted as evidence of relative deficit of cue-driven, proactive mechanisms, due to underlying systems—namely prefrontal cortices—lagging behind in development [9], being affected by age-related involution [26] or pathophysiological processes, as in schizophrenia [31]. Our results that showed protracted development well within adulthood are consistent with the trajectory of prefrontal cortex maturation outlined by anatomical studies [8]. However, AXCPT allows flexibility to occur in other dimensions. The task critically differs from standard task-switch paradigms in the implicit nature of separate task-sets and task-switch triggers. It also differs from Stop-signal, Go/No-Go and proactive inhibition paradigms as the access to the information encoded in the frequency asymmetry of trial types is accessory. These additional dimensions appear to be effectively exploited through development. Consequently, the probe-driven vs. cue-driven dichotomy does not seem to provide an exhaustive account of working memory variability in the explored age range.

The reported apparent discontinuity in maturation could be related to the developmental specificities of adolescence, but should not necessarily be interpreted as reflecting the emergence of new resources, namely the ontogeny of CP. The highlighted differences occur against a backdrop of continuous and global improvement which is underscored by similarities across ages. All participants clearly understood the task instructions, implying that errors committed by children are not related to explicitly reportable differences in the content of instructions, no more than errors at later stages of development are. Their performance can be regarded as an instance of action-knowledge dissociation which is commonly reported in development. As such it could be related to procedural conflicts that are not evident by explicit reporting. Such conflicts are different from what postulated by CP theory and are likely related to the different constraints to optimal performance posed by the asynchronously development of cognitive resources.

## Conclusions

In order to track maturation of CP from childhood to adulthood we investigated performance in an established and specific CP test, the AXCPT in a large cohort of subjects aged 8 to 22 years old. By expanding analysis beyond standard, theory-driven indices to include more data-driven approaches we were able to show that AXCPT validly track a shift from reactive strategies in adolescence to a preparatory, proactive control that appears to achieve full maturity only in early adulthood. Importantly, we also showed that standard dual mechanisms assumptions that postulate a unique reactive-proactive dimension are not met in childhood and provided a novel insight of cognitive control development.

It was previously shown that transition from infancy into childhood is accompanied by CP maturation characterized by increasing reliance on preparatory operations [9]. Studies with temporally sparse samplings in later development [10–12] are generally interpreted as a monotonic maturation in the same direction to finally achieve the proactive strategy that characterizes normal adults. All these studies relied on standard, model-based measures which when applied to our own sample suggested instead that cognitive control strategies in childhood first mature towards greater reliance on reactive operations in adolescence and then again into a more proactive control in adulthood (Fig 7). However, data-driven analyses showed fundamental differences between the proactivity observed in childhood and in adulthood and suggested that cue-driven mechanisms could be further differentiated on the basis of their control content.

Children appeared to be vulnerable to procedural conflicts not predicted by standard models and tended to rely on a preparatory strategy mainly confined to a single task-set limited to response selection. In turn, such a rudimentary strategy minimizes the impact of the conflicts that are assumed as basis for standard performance indexing limiting the interpretability of such measures. On the other hand, the strategy during early adulthood appeared to be consistent with a greater involvement of canonical control functions that extended beyond response selection to involve goal-relevant maintenance of information and cue-driven task-sets with appropriate triggering of attention shifts and proactive inhibition. It is only in this perspective that a monotonic improvement in the general direction of preparatory mechanisms maturation from infancy into early adulthood can be hypothesized.

Our study has some important limitations. It is a behavioral study and as such the biological inferences are limited and strictly dependent on the task parameters. While results are generally consistent with the role of prefrontal cortex in CP and its delayed developmental trajectory, such evidence is indirect. We explored a single set of parameters to favor comparability across a wide age range but it should be noted that there is a trade-off in developmental research between results comparability and the possibility that timing, trial type frequencies and rule understanding effects differ across age. Furthermore, we employed a cross-sectional design. Further studies are required to test explicitly whether the findings generalize across task parameters and in longitudinal designs.

We also raised a number of theoretical points concerning the interpretation AXCPT and the CP construct. It was emphasized that in a cognitive control test as AXCPT, performance improvement needs to be regarded as an optimization problem that is solved by adjusting flexibly to the available cognitive resources. As such CP maturation needs to be addressed jointly with related and subordinate functions and their specific developmental time course and it does not necessarily involve increased reliance on preparatory mechanisms. Indeed, moving from childhood into adolescence AXCPT performance improves by relying on more reactive strategies. However, such general and qualitative remarks awaits explicit testing and formal modeling.

A strength of our study relies on the use of data-driven analyses that captured differences not evident to model based indexing. The reliability of such indices depends on assumptions that need to be verified by employing more data-driven analyses. Our results suggest CP models of AXCPT performance can be reliably applied only from adolescence onwards. Accordingly, while the task paradigm can be validly used to track deviations from the reported normative trajectory its use is contingent to verification of the assumptions underlying such models. Given the cardinal role of AXCPT and standard measures in shaping the understanding of cognitive impairment in conditions such as schizophrenia and aging, the advisory remark extends beyond the relevance to developmental studies.

With these limitations in mind, we showed how a mature, proactive AXCPT performance appears to be a late acquisition of development. Starting from adolescence, early developing resources appear to become increasingly integrated into optimized performance and it is only in early adulthood that a flexible and continually updated representation of task relevant information mature. Neurodevelopmental disorders affecting such CP can alter such trajectory in different ways. For instance we showed that the reactive performance observed in adolescence resembles what described in schizophrenia. Developmental, longitudinal studies in schizophrenia patients—if paired with data-driven behavioral analysis—will be able to inform the pathophysiology of the disorder either by suggesting a delayed or an arrested maturation.

## Supporting information

S1 Table Sample description. Age bins were matched by gender, handedness, parental socioeconomic and ethnicity (68% Caucasian, 25% African-American, 4% Asian, 3% other). Sample full scale age-corrected IQ scores were balanced across age groups and in agreement with general population expectations, i.e. normally distributed (Lilliefors .078, p < .001) and centered around 100 (Z_186_ vs. 100 (±15), 3.9 p < .001). Exclusion criteria were: lifetime Axis I disorder, mental retardation, psychoactive substance dependence within the past 6 months or abuse within the past month, history of significant head injury, neurologic disorders or other medical illnesses, pregnancy, first-degree family history of psychotic disorder or mood disorder with psychotic features, or lack of capacity to provide assent or consent for participants or parents.
(TIF)

S2 Table Raw error rates and reaction times. Mean error rates and reaction times on correct responses for each age bin in each trial type. Standard deviation are reported in parenthesis.
(TIF)
