# Supplementary figures and images for: Normal development of context processing using the AXCPT paradigm
Source: PLoS One. 2018 May 31;13(5):e0197812. doi: 10.1371/journal.pone.0197812 (PMC5979008; doi:10.1371/journal.pone.0197812)

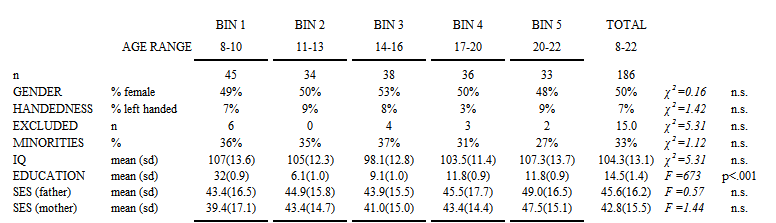

Supplement: S1 Table — Age bins were matched by gender, handedness, parental socioeconomic and ethnicity (68% Caucasian, 25% African-American, 4% Asian, 3% other). Sample full scale age-corrected IQ scores were balanced across age groups and in agreement with general population expectations, i.e. normally distributed (Lilliefors .078, p < .001) and centered around 100 (Z186 vs. 100 (±15), 3.9 p < .001). Exclusion criteria were: lifetime Axis I disorder, mental retardation, psychoactive substance dependence within the past 6 months or abuse within the past month, history of significant head injury, neurologic disorders or other medical illnesses, pregnancy, first-degree family history of psychotic disorder or mood disorder with psychotic features, or lack of capacity to provide assent or consent for participants or parents. (TIF) [file pone.0197812.s001.tif]

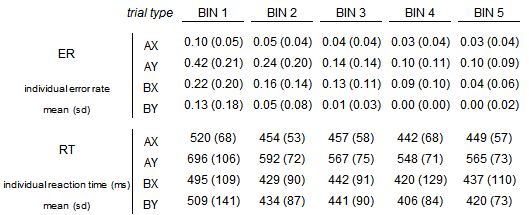

Supplement: S2 Table — Mean error rates and reaction times on correct responses for each age bin in each trial type. Standard deviation are reported in parenthesis. (TIF) [file pone.0197812.s002.tif]
